# Incidence, predictive factors and severity of methotrexate-related liver injury in rheumatoid arthritis: a longitudinal cohort study

**DOI:** 10.1093/rap/rkaa020

**Published:** 2020-06-05

**Authors:** Shunsuke Mori, Nobuyuki Arima, Masahiro Ito, Yukitaka Ueki, Yasuyo Abe, Kiyoshi Aoyagi, Shigetoshi Fujiyama

**Affiliations:** r1 Department of Rheumatology, Clinical Research Center for Rheumatic Diseases, National Hospital Organization Kumamoto Saishun Medical Center, Kohshi, Kumamoto; r2 Department of Pathology, Kumamoto Shinto General Hospital, Kumamoto; r3 Department of Pathology, Clinical Research Center, National Hospital Organization Nagasaki Medical Center, Omura, Nagasaki; r4 Rheumatic and Collagen Disease Center, Sasebo Chuo Hospital, Sasebo, Nagasaki; r5 Department of Public Health, Nagasaki University Graduate School of Biomedical Sciences, Nagasaki; r6 Department of Gastroenterology and Hepatology, Kumamoto Shinto General Hospital, Kumamoto, Japan

**Keywords:** rheumatoid arthritis, methotrexate, liver injury, pretreatment fat deposition, non-alcoholic steatohepatitis

## Abstract

**Objectives:**

The aims were to determine the incidence rate, predictive factors and severity of liver injury that develops during MTX treatment for RA and to evaluate the role of pretreatment hepatic fat deposition.

**Methods:**

We used an ongoing real-life registry containing RA patients who had started MTX between August 2007 and April 2018 at participating institutions. The liver-to-spleen attenuation ratio on CT scans at enrolment was used to evaluate pretreatment fat deposition quantitatively. Patients were followed until persistent transaminitis developed or until the end of the study. Liver biopsy was performed for patients who presented with persistent transaminitis.

**Results:**

We followed 289 new MTX users without pretreatment elevations of transaminases (mean follow-up time, 58.3 months). Hepatic fat deposition was detected in half of the patients at enrolment. During follow-up, persistent transaminitis occurred at a crude incidence rate of 3.13 per 100 person-years, and the cumulative incidence at 5 years was estimated to be 13%. A multivariate Fine–Gray regression analysis showed that the most important predictive factors were pre-existing moderate to severe fat deposition (adjusted hazard ratio, 7.69; 95% CI: 3.10, 19.10) and obesity (adjusted hazard ratio, 2.68; 95% CI: 1.37, 5.25). Non-alcoholic steatohepatitis (NASH) was the most predominant pattern in liver biopsy samples. Hepatic fibrosis was found in 90% of samples, but most cases were not advanced.

**Conclusion:**

Aggravation of underlying fatty liver to NASH with fibrosis seems to be an important mechanism of liver injury that occurs in MTX-treated RA patients.

**Key messages**

- The crude incidence rate of persistent transaminitis after MTX treatment is 3.13 per 100 person-years.
- The most important predictive factors are pretreatment moderate to severe fat deposition and obesity.
- MTX use can favour the aggravation of pre-existing fatty liver to non-alcoholic steatohepatitis with fibrosis.

## Introduction

MTX is widely used as the conventional systemic DMARD (csDMARD) of first choice to treat patients with RA, psoriatic disease and other autoimmune diseases [1–3]. With chronic MTX use, liver injury has been recognized as an important adverse event in these patients [4, 5]. Recently, the role of non-alcoholic fatty liver disease (NAFLD) as a risk factor for drug-induced liver injury has received a lot of attention [6, 7]. NAFLD is characterized by excessive hepatic fat accumulation, without significant alcohol consumption, competing aetiologies for hepatic steatosis, or coexisting causes for chronic liver disease [8–11]. Several studies reported a significant association of NAFLD risk factors, especially obesity and type 2 diabetes, with the development of histologically advanced grades of liver injury and severe fibrosis in psoriasis patients receiving MTX treatment [12–15]. In addition, the similarity of pathological features between MTX-related liver injury and a more progressive form of NAFLD, namely non-alcoholic steatohepatitis (NASH), was reported in psoriasis patients [13].

For RA patients, several studies identified obesity, type 2 diabetes and hypercholesterolaemia as risk factors for the increase in alanine and/or aspartate aminotransferase (ALT and/or AST) levels that is observed during MTX treatment [16–19]. A NASH-like pattern was reported to be a common histological abnormality in MTX-treated RA patients who had persistent transaminitis [19]. These findings suggest that use of MTX could favour aggravation of pre-existing NAFLD and progression to NASH. However, this assumption has not been proved because there is a lack of information regarding the extent of hepatic fat deposition before the start of MTX use and its effect on the development of liver injury during treatment.

To address this issue, we used an ongoing real-life registry including RA patients with no increases in transaminase levels who had commenced MTX treatment at our institution since August 2007. The liver-to-spleen attenuation ratio (L/S ratio) on high-resolution CT scans at enrolment was used to evaluate fat deposition quantitatively before the start of MTX treatment. We determined the incidence rate, predictive factors and severity of liver injury developing during MTX treatment and explored the role of pre-existing hepatic fat deposition.

## Methods

### Patients

The MET-START registry (the METhotrexate-new STARTer registry) is an ongoing real-life cohort consisting of all patients with RA who have commenced low-dose MTX treatment since August 2007 at the outpatient clinic for rheumatic diseases of National Hospital Organization (NHO) Kumamoto Saishun Medical Center in Japan. The main objective of this registry is to study the long-term safety of MTX use and to identify predictive factors for adverse events that are associated with this drug. Registrants are required to be ≥18 years of age and MTX-naïve at enrolment. They are also required to fulfil the 1987 ACR criteria or the 2010 ACR/EULAR criteria for diagnosis of RA [20, 21]. In this cohort, MTX is prescribed at 4–14 mg weekly for RA patients (a median of 8 mg/week), and 5 mg/week of folic acid is prescribed concomitantly during MTX treatment [22]. For non-elderly patients with high disease activity who are at low risk for adverse events, MTX can be started at an initial dose of 10 mg/week or more. Data from each patient at baseline and during follow-up have been deposited regularly in the Data Management Center for the MET-START registry at NHO Kumamoto Saishun Medical Center.

All participants in the present study came from the MET-START registry. We followed patients who had been enrolled in this registry during the period between 1 August 2007 and 30 April 2018. The exclusion criteria for this study were the presence of chronic liver disease, including viral hepatitis (hepatitis B and hepatitis C), autoimmune liver disease (autoimmune hepatitis and primary biliary cholangitis), hereditary liver disease and significant ethanol intake (>30 g/day for males and >20 g/day for females) at the time of starting MTX treatment. In addition, eligible patients were required to have normal serum ALT and AST levels at MTX initiation.

### Study design

To identify patients who had developed persistent transaminitis, we monitored participant serum ALT and AST levels at each visit (i.e. every 4–8 weeks) during MTX treatment. Persistent transaminitis was defined as elevations in ALT and/or AST levels above the upper limit of normal in five of nine determinations within a given 12 month interval (6 of 12 determinations if tests are performed monthly) [23]. The upper limit of normal value for ALT and AST that was used in this study was 30 IU/l. Follow-up started on the first day of MTX treatment and ended with the development of persistent transaminitis, MTX discontinuation, loss to follow-up, death or the last follow-up visit before 30 April 2019, whichever was first. MTX discontinuation was defined as no use of this drug for >3 months. The reasons for MTX discontinuation included adverse events, inefficacy and other reasons (e.g. hospital transfer, surgery). Patients who missed at least two scheduled visits without any contact were classified as lost to follow-up.

### Patient characteristics at the start of MTX use

For each patient, demographic characteristics and RA-related factors, such as RA duration, Steinbrocker’s radiological stage, serum CRP levels, ESR values, positivity of anti-CCP antibodies and RF, together with the presence of NAFLD risk factors and co-morbidities, including hypertension, type 2 diabetes, chronic kidney disease (CKD), smoking history and BMI, were examined at enrolment in the MET-START registry. The definitions of hypertension, type 2 diabetes and CKD were given elsewhere [24]. Concurrent use of prednisolone, NSAIDs and other DMARDs was recorded at the same time. In addition, the use of hepatotoxic drugs apart from RA medications was examined.

In the NHO Kumamoto Saishun Medical Center, a high-resolution CT examination extending from the lung apices to the diaphragm is performed on RA patients who are scheduled to receive MTX, because it is essential to evaluate pulmonary conditions carefully. This policy makes it possible to evaluate quantitatively the fat infiltration in the liver of unselected RA patients before starting MTX treatment. The L/S ratio on high-resolution CT imaging was calculated on a SYNAPSE EX workstation (Fujifilm, Tokyo, Japan). Hepatic and splenic attenuation values were measured on unenhanced high-resolution CT scans using four circular region-of-interest cursors in the liver (two in the right lobe and two in the left lobe) and three in the spleen. Average attenuation values of the liver and spleen were calculated and used to determine the L/S ratio. Based on L/S ratio data from previous studies with Japanese individuals, we interpreted an L/S ratio of ≥1.3 to indicate no steatosis. To differentiate between mild (<30%) and moderate to severe (≥30%) steatosis, we set a cut-off level for the L/S ratio of 1.1, as follows: L/S ratio of ≥1.1 and <1.3 was defined as mild steatosis and L/S ratio of <1.1 as moderate to severe steatosis [25, 26].

### Assessment of patients who developed persistent transaminitis after the start of MTX use

For radiological evaluation of the liver in patients who developed persistent transaminitis during MTX treatment, we performed abdominal US and high-resolution CT without reference to any of the patients’ clinical data. A radiological diagnosis of fatty liver was made based on the following four abnormal findings on US scan: bright liver, hepatorenal echo contrast, vascular blurring and deep attenuation [27]. Hepatic fibrosis was identified based on the following morphological abnormalities: irregular or nodular liver surface, coarse or non-homogeneous liver parenchymal echotexture and blunted or rounded liver edge [28]. We also measured autoantibodies [ANA and anti-mitochondrial M2 antibody (AMA-M2)] and serological markers for hepatitis B and C.

For assessment of the type and severity of liver injury, US-guided liver biopsy was performed for patients who developed persistent transaminitis. Patients who had contraindications to liver biopsy and those who refused to undergo this procedure were excluded. Histological assessment of the type and severity of fatty liver were performed in accordance with the following two pathological criteria: the NASH Clinical Research Network pathological criteria (NAS; NAFLD activity score) [29] and the Younossi criteria (revised version of the Matteoni criteria) [30, 31]. Histological parameters were evaluated for each liver biopsy specimen independently by two board-certified experts in liver pathology (N.A. and M.I.). Both observers were blinded to the patients’ clinical status, and the final diagnosis was determined by consensus. NASH was diagnosed based on the following criteria: (a) any degree of steatosis along with centrilobular ballooning of hepatocytes and/or Mallory–Denk bodies; or (b) any degree of steatosis along with centrilobular pericellular/perisinusoidal fibrosis or bridging fibrosis, if patients had no identifiable secondary causes of hepatic fat accumulation [31]. Steatosis alone or steatosis with lobular inflammation was considered to be non-NASH NAFLD (non-alcoholic fatty liver: NAFL) [30]. Staging of fibrosis was based on the NAS system. Advanced fibrosis was defined as NAS staging 3 and 4.

This study was conducted in accordance with the principles of the Declaration of Helsinki (2008). The protocol of this study also meets the requirements of the Ethical Guidelines for Medical and Health Research Involving Human Subjects, Japan (2014) and has been approved by the Human Research Ethics Committee of NHO Kumamoto Saishun Medical Center (no. 28-06). Informed written consent was obtained from all participants.

### Statistical analysis

Crude incidence rates of persistent transaminitis and the 95% CI were calculated by dividing the number of incidence cases by the number of corresponding follow-up person-years overall and for each patient group.

The probability of persistent transaminitis over time was computed using the cumulative incidence function (CIF), because we considered the presence of competing risks. Gray’s test was used to compare the estimates between each patient group.

Fine–Gray competing risks regression analysis was used to evaluate the effect of each patient characteristic on the development of persistent transaminitis during MTX treatment and to calculate adjusted hazard ratios (HRs), treating MTX discontinuation, death and loss to follow-up as competing events. As predictor variables, we used baseline patient characteristics that were considered to be clinically relevant variables. We first performed univariate Fine–Gray regression analysis for each of the predictor variables. Thereafter, all variables with *P*-values <0.20 in univariate models were introduced into a multivariate Fine–Gray regression analysis. The proportional hazards assumption was checked using log-minus-log plots of log cumulative hazard curve function and scaled Schoenfeld residual plots for exposure variables over time. By calculating the variance inflation factor, we confirmed that there was no multicollinearity among predictor variables.

For all tests, the probability values (*P*-values) <0.05 were considered to indicate statistical significance. All calculations were performed using PASW Statistics v.22 (SPSS Japan Inc., Tokyo, Japan) and Easy R (Saitama Medical Center, Jichi Medical University, Saitama Japan) [32].

## Results

### Baseline patient characteristics

There were 289 patients who commenced MTX treatment (a median of 8 mg/week) for RA and were enrolled into this study. Baseline characteristics are shown in Table 1. Most cases were early RA (disease duration <2 years, 79.9%). NSAIDs and prednisolone were prescribed in 32.3 and 28.0% of patients, respectively. Sixty-four patients (22.1%) were obese (BMI ≥25 kg/m^2^), and 6.2% had type 2 diabetes. Approximately half of the patients had fat deposition, as evidenced by the L/S ratio on high-resolution CT scans (42.6% with mild fat deposition and 5.9% with moderate to severe fat deposition). No patients used acetaminophen or other hepatotoxic drugs except RA medications.

**Table 1:** Patient characteristics at the time of first starting MTX use for RA (*n* = 289)

Characteristic	Entire cohort
Age, years, mean (95% CI)	60.4 (59.0, 61.7)
Male/female, *n*	70/219
MTX weekly dose, mg, median (95% CI)	8.4 (8.2, 8.6)
RA duration, months, mean (95% CI)	23.2 (15.7, 30.7)
<2 years (early RA), *n* (%)	231 (79.9)
Anti-CCP positive, *n* (%)	263 (91.0)
RF positive, *n* (%)	238 (82.4)
Steinbrocker’s stages III/IV, *n* (%)	52 (18.0)
CRP, mg/dl, mean (95% CI)	1.71 (1.42, 2.00)
≥1.5 mg/dl^a^, *n* (%)	95 (32.9)
ESR, mm/h, mean (95% CI)	37.7 (34.6, 40.9)
≥28 mm/h^a^, *n* (%)	169 (58.5)
L/S ratio on high-resolution CT, mean (95% CI)	1.29 (1.27, 1.30)
≥1.3 (no fat deposition), *n* (%)	149 (51.6)
≥1.1 and <1.3 (mild fat liver), *n* (%)	123 (42.6)
<1.1 (moderate to severe fat liver), *n* (%)	17 (5.9)
BMI, kg/m^2^, mean (95% CI)	22.6 (22.2, 23.1)
≥25 (obesity), *n* (%)	64 (22.1)
Hypertension, *n* (%)	88 (30.4)
Type 2 diabetes, *n* (%)	18 (6.2)
Chronic kidney disease, *n* (%)	41 (14.2)
Current/ex-smokers^b^, *n* (%)	96 (33.2)
Concurrent use of other RA medications	
NSAIDs, *n* (%)	93 (32.2)
Prednisolone, *n* (%)	81 (28.0)
bDMARDs, *n* (%)	12 (4.2)
csDMARD^c^, *n* (%)	5 (1.7)
tsDMARD^c^, *n* (%)	0

Data were obtained at the time of enrolment in the MET-START registry.

a Cut-off values were set based on the inclusion criteria often used for clinical trials of new DMARDs.

b Defined as current or former smokers with a smoking history ≥10 pack-years.

c Tacrolimus was used as a csDMARD, and no tsDMARD was used concurrently with MTX. bDMARDs: biological DMARDs; csDMARD: conventional systemic DMARD; L/S ratio: liver-to-spleen attenuation ratio; tsDMARD: targeted synthetic DMARD.

### Incidence of persistent transaminitis during MTX treatment

**Fig. 1:**
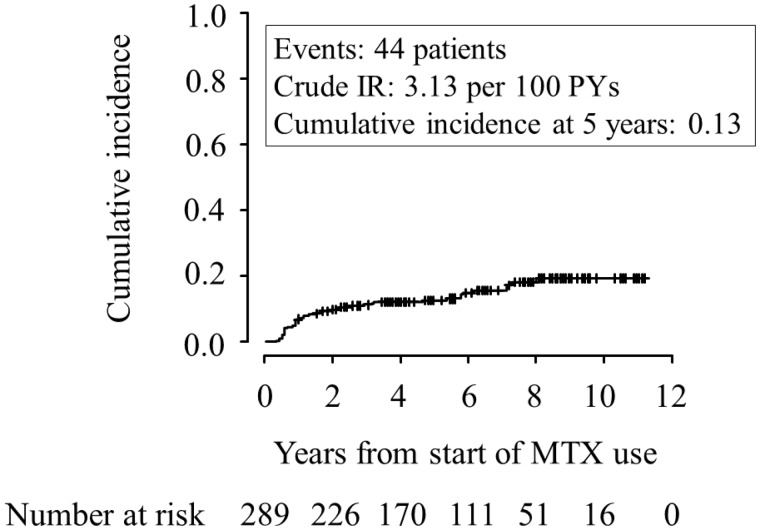
Cumulative incidence of persistent transaminitis during MTX treatment for RA Using the CIF, the cumulative incidence of persistent transaminitis occurring in RA patients who initially started MTX treatment is shown. Numbers below these figures represent the number of patients remaining on MTX treatment. CIF: cumulative incidence function; IR: incidence rate; PYs: person-years

Serum ALT and AST levels were monitored over follow-up periods, with a mean follow-up time of 58.3 months (95% CI: 54.2, 62.5). Persistent transaminitis was found in 44 patients (15.2%) over 1405 person-years at risk. The crude incidence rate was 3.13 per 100 person-years (95% CI: 2.33, 4.21). During follow-up, 70 patients discontinued MTX because of adverse events (7 patients), inefficacy (36 patients) and hospital transfer (27 patients). Adverse events included aggravation of interstitial pneumonia (three patients), cholangiocarcinoma (one patient), oesophageal carcinoma (one patient), *Pneumocystis jirovecii* pneumonia (one patient) and myocardial infarction (one patient). Among the adverse event cases, three patients with cholangiocarcinoma, *Pneumocystis jirovecii* pneumonia or myocardial infarction eventually died. No other patients died. Fifteen patients (5.0%) were categorized as lost to follow-up. According to CIF analysis based on a competing risks model, the cumulative incidence (95% CI) of persistent transaminitis at 2, 5 and 7 years was 0.10 (0.07, 0.14), 0.13 (0.09, 0.17) and 0.16 (0.11, 0.21), respectively (Fig. 1).

### Predictive factors for the development of persistent transaminitis during MTX treatment

Results of univariate and multivariate Fine–Gray competing risks regression analyses are shown in Table 2. Based on the results of univariate analyses, all variables with *P*-values <0.20 were included in multivariate analysis. Through multivariate modelling, moderate to severe fat deposition (L/S ratio <1.1), obesity, type 2 diabetes and prednisolone use at baseline were identified as significant predictive factors for persistent transaminitis. There was no multicollinearity among these variables. Among them, a baseline L/S ratio <1.1 and obesity were particularly important predictive factors for persistent transaminitis; the adjusted HR (95% CI) was 7.69 (3.10, 19.10) for L/S ratio <1.1 *vs* L/S ratio ≥1.3 (*P *<* *0.001) and 2.68 (1.37, 5.25) for obesity *vs* non-obesity (*P *=* *0.004). Other RA-related factors or co-morbid conditions did not remain in the final multivariate regression model.

**Table 2:** Predictive factors for developing persistent transaminitis during MTX treatment for RA

Variables at baseline	Unadjusted HR (95% CI)	*P*-value	Adjusted HR (95% CI)	*P*-value
Age per 1 year more	1.00 (0.98, 1.02)	0.98	–	–
Male *vs* female	0.68 (0.32, 1.44)	0.31	–	–
Weekly dose of MTX per 1.0 mg more	1.11 (0.96, 1.29)	0.16	–	–
RA duration per 1 month more	1.00 (1.00, 1.00)	0.38	–	–
Anti-CCP positive	2.04 (0.50, 8.39)	0.32	–	–
RF positive	0.80 (0.38, 1.67)	0.55	–	–
Steinbrocker’s stages III/IV *vs* I/II	1.39 (0.72, 2.68)	0.33	–	–
CRP ≥1.5 mg/dl	1.33 (0.73, 2.42)	0.36	–	–
ESR ≥28 mm/h	1.96 (1.00, 3.89)	0.051	–	–
L/S ratio on high-resolution CT				
≥1.3 (no fat deposition)	Reference	–	Reference	–
≥1.1 and <1.3 (mild fat deposition)	1.88 (0.95, 3.74)	0.072	1.85 (0.94, 3.67)	0.070
<1.1 (moderate to severe fat deposition)	10.76 (4.75, 24.37)	<0.001	7.69 (3.10, 19.10)	<0.001
Obesity (BMI ≥25 kg/m^2^)	3.50 (1.94, 6.33)	<0.001	2.68 (1.37, 5.25)	0.004
Hypertension	1.49 (0.83, 2.67)	0.18	–	–
Type 2 diabetes	2.25 (0.87, 5.81)	0.10	2.76 (1.02, 7.48)	0.046
Chronic kidney disease	0.55 (0.20, 1.51)	0.24	–	–
Current/ex-smokers	0.65 (0.33, 1.28)	0.21	–	–
NSAID use	1.51 (0.84, 2.72)	0.17	–	–
Prednisolone use	2.01 (1.11, 3.64)	0.020	1.96 (1.09, 3.51)	0.030
bDMARD use	0.38 (0.05, 2.86)	0.34	–	–

Univariate and multivariate Fine–Gray competing risks regression analyses were conducted to evaluate baseline patient-specific factors that predict the development of persistent transaminitis during MTX treatment. All variables with *P*-values <0.20 in the univariate Fine–Gray models were introduced into multivariate analysis. Variables that remained in the final multivariate model are shown as significant predictive factors for persistent transaminitis.

bDMARDs: biological DMARDs; HR: hazard ratio; L/S ratio: liver-to-spleen attenuation ratio.

**Fig. 2:**
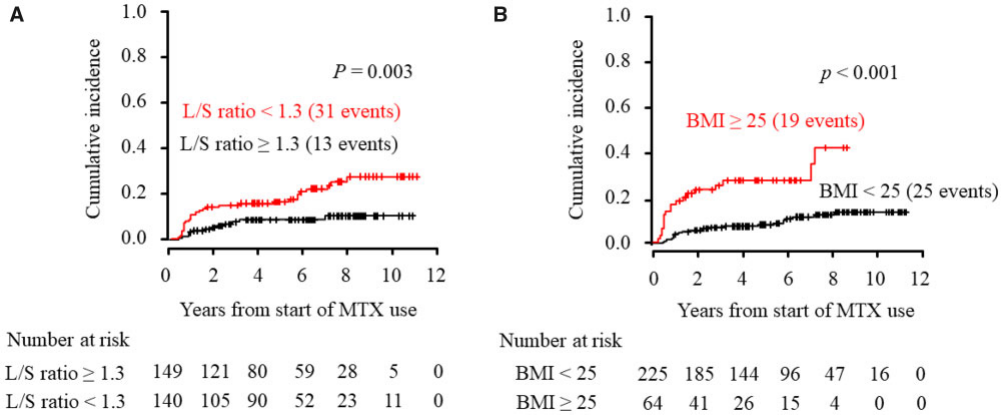
Cumulative incidence of persistent transaminitis grouped by predictive factors Using the CIF, the cumulative incidence of persistent transaminitis occurring in RA patients who initially started MTX treatment are shown grouped according to predictive factors for persistent transaminitis. Predictive factors included (**A**) the pre-existence of fat deposition (L/S ratio <1.3) and (**B**) obesity (BMI ≥25 kg/m^2^). Numbers below these figures represent the number of patients remaining on MTX treatment. Provability of survival without persistent transaminitis between the groups with and without predictive factors was compared using Gray’s test. CIF: cumulative incidence function; L/S ratio: liver-to-spleen attenuation ratio.

Using CIF analysis with Gray’s test, the cumulative incidence of persistent transaminitis during MTX treatment was compared between patients with and without the presence of hepatic fat deposition at baseline (L/S ratio <1.3 *vs* L/S ratio ≥1.3; Fig. 2A) and between those with and without obesity (BMI ≥25 *vs* BMI <25 kg/m^2^; Fig. 2B). The cumulative incidence of persistent transaminitis in patients with fat deposition at baseline was significantly higher over time compared with those without this condition (*P *=* *0.003). Likewise, obese patients had a higher cumulative incidence than non-obese patients (*P *<* *0.001).

The crude incidence rate in patients with and without hepatic fat deposition at baseline was 4.50 per 100 person-years (95% CI: 3.20, 6.41) and 1.81 per 100 person-years (95% CI: 1.10, 3.10), respectively. The crude incidence rate was 8.20 per 100 person-years (95% CI: 5.20, 12.91) in obese patients and 2.10 per 100 person-years (1.40, 3.21) in non-obese patients.

### Assessment of patients who developed persistent transaminitis during MTX treatment

As shown in Table 3, the median time of MTX use to the diagnosis of persistent transaminitis was 29.0 months (a median cumulative MTX dose of 1113 mg). According to the L/S ratio, moderate to severe fat deposition had already existed in 22.7% of patients at the start of MTX treatment. At the development of persistent transaminitis, the rate of patients with moderate to severe fat deposition was 45.5%. According to US examinations, 31 out of 44 patients (70.5%) showed fatty liver (43.2% with fatty liver alone and 27.3% with fatty liver plus fibrosis). These patients had no secondary causes of steatosis, including alcoholic fatty liver, chronic viral hepatitis, autoimmune liver disease or hereditary liver disease. Seven patients were diagnosed with hepatic fibrosis and six patients had no abdominal US findings. One patient without abnormal US findings was ultimately diagnosed with primary biliary cholangitis.

**Table 3:** Assessment of patients who developed persistent transaminitis during MTX treatment for RA (*n* = 44)

Characteristic	At baseline	At development
Age, years, mean (95% CI)	60.5 (57.8, 61.7)	63.0 (60.2, 65.8)
Male/female, *n*	8/36	‒
MTX use		
Weekly dose, mg, median (95% CI)	8.7 (8.1, 9.2)	8.7 (8.1, 9.3)
Duration, months, median (95% CI)	0	29.0 (20.5, 37.6)
Cumulative dose, mg, median (95% CI)	0	1113 (750, 1476)
US findings		
Fatty liver	‒	19 (43.2)
Fatty liver + hepatic fibrosis	–	12 (27.3)
Hepatic fibrosis	‒	7 (15.9)
No abnormal findings	‒	6 (13.6)
L/S ratio on high-resolution CT, mean (95% CI)	1.20 (1.14, 1.25)	1.14 (1.09, 1.20)
≥1.3 (no fat deposition), *n* (%)	13 (29.5)	9 (20.5)
≥1.1 and <1.3 (mild fat deposition), *n* (%)	21 (47.7)	15 (34.1)
<1.1 (moderate to severe fat deposition), *n* (%)	10 (22.7)	20 (45.5)
BMI, kg/m^2^, mean (95% CI)	24.8 (23.5, 26.1)	24.7 (23.4, 26.1)
≥25 (obesity), *n* (%)	19 (43.2)	18 (40.9)
Hypertension, *n* (%)	18 (40.9)	20 (45.5)
Type 2 diabetes, *n* (%)	5 (11.4)	7 (15.9)
Chronic kidney disease, *n* (%)	4 (9.1)	5 (11.4)
Concurrent use of other RA medications		
NSAIDs, *n* (%)	19 (43.2)	10 (22.7)
Prednisolone, *n* (%)	19 (43.2)	5 (11.4)
Autoantibodies		
ANA titres ≥1:160, *n* (%)	–	2 (4.5)
AMA-M2 positive, *n* (%)	–	1 (2.3)

Data were obtained when MTX use began (at baseline) and when persistent transaminitis developed (at development).

AMA-M2: anti-mitochondrial M2 antibody; L/S ratio: liver-to-spleen attenuation ratio.

### Liver biopsy

Out of 44 patients who presented with persistent transaminitis, 24 underwent liver biopsy (Table 4). Among them, 19 (79.2%) and three (12.5%) were classified as having NASH and NAFL, respectively. They included 19 cases of US-diagnosed fatty liver (7 with fatty liver alone and 12 with fatty liver plus hepatic fibrosis), one case of hepatic fibrosis and two cases without abnormal US findings. Interface hepatitis was observed in the two other patients, who were also diagnosed radiologically with hepatic fibrosis. The remaining 20 patients did not receive liver biopsy (8 owing to advanced age, one with a diagnosis of primary biliary cholangitis and 11 who refused to undergo the procedure). This group consisted of 12 patients with US-diagnosed fatty liver alone, 4 with hepatic fibrosis and 4 without abnormal US findings. There were no significant differences in ALT or AST levels between patients receiving biopsy and those who did not [mean ALT: 20.1 (95% CI: 16.0, 24.2) *vs* 23.5 IU/l (95% CI: 18.9, 28); mean AST: 20.2 (95% CI: 18.2, 22.3) *vs* 21.8 IU/l (18.8, 24.8)].

**Table 4:** Type and severity of fatty liver: data from liver biopsy (*n* = 24)

Fatty liver	At development
Histological classification, *n* (%)	
NASH	19 (79.2)
NAFL	3 (12.5)
Interface hepatitis	2 (8.3)
NAS fibrosis staging, *n* (%)	
Stage 0 (no fibrosis)	3 (12.5)
Stage 1 (perisinusoidal or periportal fibrosis)	11 (45.8)
1A (mild, zone 3, perisinusoidal fibrosis)	8 (33.3)
1B (moderate, zone 3, perisinusoidal fibrosis)	1 (4.2)
1C (portal/periportal fibrosis)	2 (8.3)
Stage 2 (perisinusoidal plus periportal fibrosis)	5 (20.8)
Stage 3 (bridging fibrosis)	5 (20.8)
Stage 4 (cirrhosis)	0

Data were obtained when persistent transaminitis developed (at development).

NAFL: non-alcoholic fatty liver; NAFLD: non-alcoholic fatty liver disease; NASH: non-alcoholic steatohepatitis; NAS: NAFLD activity score.

Hepatic fibrosis was observed in 21 patients (87.5%), comprising 19 cases of NASH and 2 cases of interface hepatitis. Among them, only five patients (four cases of NASH and one case of interface hepatitis) had bridging fibrosis. No cirrhosis was observed.

## Discussion

In this register-based RA cohort study, persistent transaminitis occurred at a crude incidence rate of 3.13 per 100 person-years in new MTX users who did not have increased pretreatment transaminase levels. The cumulative incidence at 5 years was estimated to be 13%. The most important predictive factors were the pre-existence of moderate to severe fat deposition and obesity at baseline. Approximately 70% of patients who had developed persistent transaminitis had fatty liver without competing aetiologies for chronic liver disease. NASH was the most predominant histological pattern in liver biopsy samples. Hepatic fibrosis was found in nearly 90% of biopsy samples, but most cases were not advanced.

Although the association between NAFLD risk factors and liver injury during MTX treatment for inflammatory rheumatic diseases has been reported [12–19], information about the extent of pretreatment fat deposition in the liver and its effect on the development of liver injury is lacking. In the present study, 5.9% of MTX-naïve RA patients without pretreatment elevations of transaminases had moderate to severe fat deposition. Given that all participants in this study had no competing aetiologies for hepatic steatosis at enrolment, these patients were considered to have NAFLD before starting MTX treatment. We showed that the pre-existence of moderate to severe fat deposition was a significant predictive factor for the development of liver injury during MTX treatment for RA. In liver biopsies for patients who had developed persistent transaminitis, the NASH pattern was the most prevalent histological pattern, and all patients with the NASH pattern had hepatic fibrosis. These findings support the notion that the aggravation of pre-existing NAFLD lesions is an important mechanism of liver injury that occurs after MTX treatment for RA. Continuous use of MTX can favour the worsening of underlying NAFLD to NASH with fibrosis.

In the present study, hepatic fibrosis was commonly observed in biopsy samples, but advanced fibrosis (NAS fibrosis scores ≥3) was found in only one-fifth of the specimens. No patients showed cirrhosis in liver biopsies. This might be explained by the fact that liver biopsy was performed at the time of first development of persistent transaminitis (a mean cumulative MTX dose of 1.1 g). Aithal *et al.* [33] reported that the cumulative amount of MTX influences the severity of liver fibrosis in psoriasis patients. In that study, the frequency of advanced fibrosis (Ishak scores ≥4) in MTX-treated patients increased from 0% at a cumulative MTX dose of 1.5 g to 2.6% at 3 g, 2.6% at 4.5 g and 8.2% at 5 g. Conversely, Rosenberg *et al.* [15] showed that psoriasis patients with risk factors, especially obesity or type 2 diabetes, are at higher risk of developing advanced liver fibrosis (NAS scores ≥3) than those without any pre-existing risk factors, even when lower cumulative MTX doses are given. In that study, 38% of patients with risk factors had advanced fibrosis at a median cumulative dose of 1.6 g compared with 9% without any risk factors at a median dose of 1.9 g. A study of liver transplant recipients in the USA showed that end-stage MTX-related liver disease is very rare among these patients [34].

There are several limitations to this study. First, we could not perform liver biopsy for all patients who had developed persistent transaminitis during MTX treatment, because there is no strong recommendation for a liver biopsy for MTX-treated RA patients [35]. Non-invasive methods for the diagnosis of hepatic fibrosis and cirrhosis, such as transient elastography and shear wave elastography, might be considered in future studies that assess the severity of hepatic fibrosis in MTX-treated RA patients [36, 37]. Second, we did not include MTX-untreated patients as controls. A follow-up study including such controls would be helpful in exploring a causal relationship between MTX use and worsening of underlying NAFLD lesions. However, it is ethically unacceptable, because the current guidelines recommend that DMARD therapy be started as soon as the diagnosis of RA is made and that MTX is the first choice for RA treatment [3]. The present study was performed using an inception cohort of MTX, and all patients had no increases in hepatic enzymes at baseline. In addition to information on NAFLD risk factors, the quantitative data on pretreatment fat deposition in the liver were available in all patients. The present study, therefore, provides important information to answer the question of whether persistent transaminitis in MTX-treated patients might be related to a new occurrence of steatohepatitis or aggravation of pre-existing NAFLD lesions.

In conclusion, pre-existing moderate to severe fat deposition in the liver and obesity are the most potent predictive factors for persistent transaminitis during MTX treatment for RA. The aggravation of underlying fatty liver to NASH with fibrosis seems to be the important mechanism in the development of liver injury in MTX-treated patients. Considering that fat deposition was detected in half of MTX-naïve RA patients at enrolment in this registry, careful and regular monitoring of transaminases is required during MTX treatment. Quantitative evaluation of pretreatment fat deposition in the liver is useful to identify patients who are at high risk for developing a liver injury during MTX treatment.
